# Successful treatment of periprosthetic joint infection caused by *Granulicatella para-adiacens* with prosthesis retention: a case report

**DOI:** 10.1186/s12891-016-1008-9

**Published:** 2016-04-12

**Authors:** Nora Renz, Fabienne Chevaux, Olivier Borens, Andrej Trampuz

**Affiliations:** Center for Septic Surgery/Infectious Diseases, Charité - University Medicine Berlin, Charitéplatz 1, D-10117 Berlin, Germany; Department of Medicine, Lausanne University Hospital, Rue du Bugnon 46, CH-1011 Lausanne, Switzerland; Division of Orthopedic Surgery and Traumatology, Lausanne University Hospital, Rue du Bugnon 46, CH-1011 Lausanne, Switzerland

**Keywords:** *Granulicatella*, Nutritionally variant streptococci, Prosthetic joint infection, Rifampin, Endocarditis, Prosthesis retention

## Abstract

**Background:**

*Granulicatella* and *Abiotrophia* spp. are difficult to detect due to their complex nutritional requirements. Infections with these organisms are associated with high treatment failure rates. We report the first implant-associated infection caused by *Granulicatella para-adiacens*, which was cured with anti-microbial treatment consisting of anti-biofilm-active rifampin and debridement, exchange of mobile parts and retention of the prosthesis.

**Case presentation:**

Patient with a history of left hip arthroplasty presented with acute onset of fever, pain and limited range of motion of the left hip. Arthrocentesis of the affected joint yielded purulent fluid and exchange of mobile parts of the prosthesis, but retention of fixed components was performed. *Granulicatella para-adiacens* grew from preoperative and intraoperative cultures, including sonication fluid of the removed implant. The transesophageal echocardiography showed a vegetation on the mitral valve; the orthopantogram demonstrated a periapical dental abscess. The patient was treated with intravenous penicillin G and gentamicin for 4 weeks, followed by levofloxacin and rifampin for additional 2 months. At discharge and at follow-up 1, 2 and 5 years later, the patient was noted to have a functional, pain-free, and radiologically stable hip prosthesis and the serum C-reactive protein was normal.

**Conclusions:**

Although considered a difficult-to-treat organism, we report a successful treatment of the *Granulicatella* hip prosthesis infection with prosthesis retention and a prolonged antibiofilm therapy including rifampin. The periapical dental abscess is considered the primary focus of hematogenously infected hip prosthesis, underlining the importance treatment of periodontitis prior to arthroplasty and of proper oral hygiene for prevention of hematogenous infection after arthroplasty.

## Background

The genera *Granulicatella* and *Abiotrophia*, formerly known as “nutritionally variant streptococci” (NVS) are part of normal oral, urogenital and intestinal flora in humans [1, 2]. They are rarely reported as human pathogens. They can cause infective endocarditis, comprising 4.3-6 % of endocarditis and other endovascular infections [3–7], meningitis, osteoarticular infections, endophthalmitis, keratitis and chronic sinusitis [8–11]. Due to common treatment failure in endocarditis and osteoarticular infection, NVS are considered difficult-to-treat organisms with frequent treatment failures [12–14]. Particularly in infective endocarditis relapse rates up to 41 % were reported [2, 3, 15]. Among implant-associated infections, only five cases of periprosthetic joint infections with *Granulicatella* or *Abiotrophia* spp. [12, 16–19] and one breast implant infection with *Granulicatella adiacens* have been described [20].

Since the first description of NVS by Frenkel and Hirsch in 1961 when isolated from a patient with infective endocarditis and otitis media [21], the terminology of NVS has changed several times. The species *Abiotrophia para-adiacens* was proposed on the basis of 16sRNA analysis [22]. Finally, reclassification of *Abiotrophia adiacens*, *balaenopterae* and *elegans* to a new genus *Granulicatella* was proposed in 2000 [23]. These gram-positive cocci or coccobacilli are nutritionally deficient, slow-growing and fastidious, expressing better growth around colonies of other bacteria, a microbiological phenomenon known as satellitism [14, 24]. They require blood or hemoglobin, amino acids and some vitamins to grow. These complex nutritional requirements make their detection often difficult. Because of their unique culture requirements and pleomorphic phenotypic features, misidentification or nonidentification by using commercially available phenotypic testing occur, prompting the recommendation that 16S rRNA gene sequencing be used for species-level identification of NVS infections. The Clinical and Laboratory Standards Institute (CLSI) does not recommend disk diffusion testing to determine the antimicrobial susceptibility of NVS and suggests broth microdilution MIC (minimal inhibitory concentration) testing in cation-adjusted Mueller-Hinton broth with 2.5 %–5 % lysed horse blood and 0.001 % pyridoxine hydrochloride [25].

The optimal antimicrobial treatment of NVS is not defined, especially data on implant-associated infections are scarce [12, 16–20]. In vitro antimicrobial drug susceptibility patterns do not correlate well with clinical response to treatment, and infections with NVS often respond poorly to antimicrobial treatment. Higher rates of microbiological failure and relapse rates after treatment have been observed for infection with NVS than with streptococci and related genera [2, 3, 12, 13, 15]. No experimental data are available regarding the activity of rifampin against *Granulicatella* or *Abiotrophia* spp. biofilms. Nevertheless, rifampin was used in several cases including infection of the breast implant [20], cardiac pacemaker and vertebral osteomyelitis [26] and knee periprosthetic joint infection [12]. No resistance to rifampin has been reported in NVS [24].

## Case presentation

A 65-years-old man was admitted to hospital with a 3-day history of acute left hip pain accompanied by fever, chills and malaise. A non-cemented metal-on-metal hip prosthesis was implanted on the left side after a traumatic fracture 7 years ago. The postoperative course after arthroplasty was unremarkable and the joint function was satisfactory until the recent event. At admission, the patient was febrile (38.7 °C), complaining about hip pain at mobilization with a limited range of motion. No redness, swelling, warmth or sinus tract was observed. A severe periodontitis was noticed by oral examination. Heart and lung auscultation was normal, abdominal examination was unremarkable, no skin or mucosal abnormalities were observed. At admission leukocyte count was 8.5 G/L (normal 4-10 G/L), platelet count 190 G/L (normal 150-300 G/L) and creatinine 96 mmol/L (normal 62-106 mmol/L). Liver transaminases were normal, serum C-reactive protein was 262 mg/L (normal <10 mg/L) and rheumatoid factor was negative (11 U/mL, normal <20 U/mL). Ultrasonography of the left hip showed joint effusion and 4 mL of purulent synovial fluid was aspirated. The synovial leukocyte count was 12,500 cells/μL with 92 % granulocytes. No microorganisms were seen in the Gram stain.

**Fig. 1:**
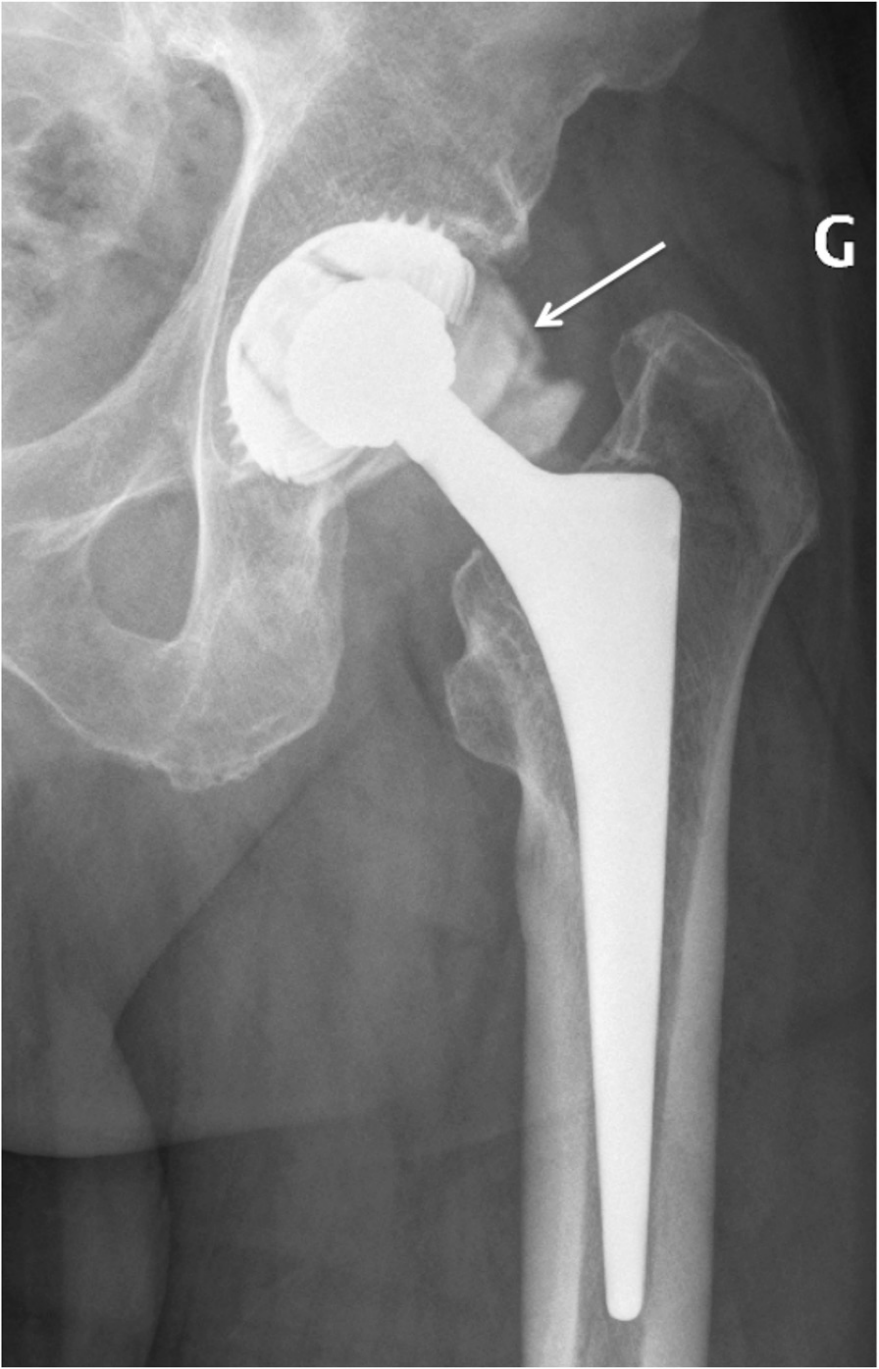
Preoperative conventional x-ray of the hip prosthesis, imaging with intra-articular contrast medium injection (arrow)

Conventional hip x-ray showed no signs of prosthesis loosening (Fig. 1). A revision hip surgery was performed on the day after admission with exchange of mobile parts (polyethylene part and head of the prosthesis) and extensive débridement, but retention of the femoral and acetabular prosthesis components. Removed mobile parts were sent for microbiological investigation for adherent biofilms by sonication. During surgery, purulent fluid was discharged after opening the joint capsule. Empiric treatment with intravenous amoxicillin/clavulanic acid (2.2 g every 8 h) was initiated.

Synovial fluid aspirate, periprosthetic tissue and sonication fluid were cultured aerobically and anaerobically for 14 days. A microorganism subsequently identified as *Granulicatella para-adiacens* grew from the sonication fluid on day 3 (>1000 colony-forming units/mL fluid), from the hip aspirate on day 9 and from one of three intraoperatively collected periprosthetic tissue samples on day 10. Blood cultures remained negative during 14 days of incubation. The identification of *Granulicatella* spp. was performed by biochemical tests and sequencing determination based on the 16S rRNA technology [27]. The minimal inhibitory concentration (MIC), performed by E-test (AB Biodisk, Solna, Sweden), was 0.19 mg/l for benzylpenicillin, 1 mg/L for ceftriaxone, 0.002 mg/L for rifampin and 0.25 mg/L for levofloxacin. According to CLSI interpretive criteria [25], the *Granulicatella para-adiacens* isolate was intermediate susceptible to penicillin (susceptible at MIC ≤0.12 mg/L), just below the susceptible breakpoint of ceftriaxone (susceptible at MIC ≤1 mg/L). No interpretive criteria are established by the CLSI for levofloxacin and rifampin, but the MIC values were low.

**Fig. 2:**
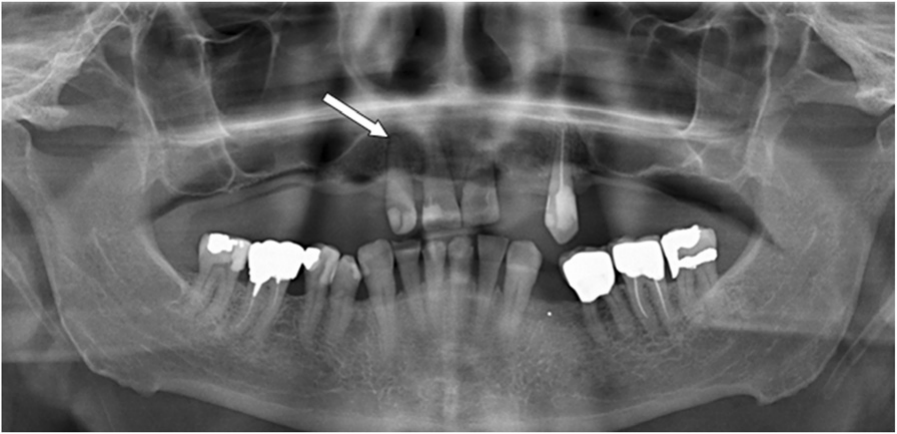
Orthopantogram at hospital admission showing severe periodontitis and periapical abscess of tooth Nr. 12 (arrow). The dental abscess is the most likely origin of hematogenous seeding of *Granulicatella* spp. to the hip prosthesis. An extraction of the tooth was performed during intravenous antibiotic treatment

The empiric therapy was modified on day 3 to intravenous benzylpenicillin (5 million units every 6 h) plus gentamicin (80 mg every 8 h) for 4 weeks. Transesophageal echocardiography demonstrated a non-mobile vegetation on the posterior leaflet of the mitral valve (size 4 x 6 mm). Transesophageal echocardiography was repeated 4 weeks later and the size of the vegetation remained unchanged. In the orthopantogram, 15 missing teeth and a periapical dental abscess of tooth No. 12 was noticed (Fig. 2). Tooth extraction was performed during the intravenous antibiotic therapy.

**Fig. 3:**
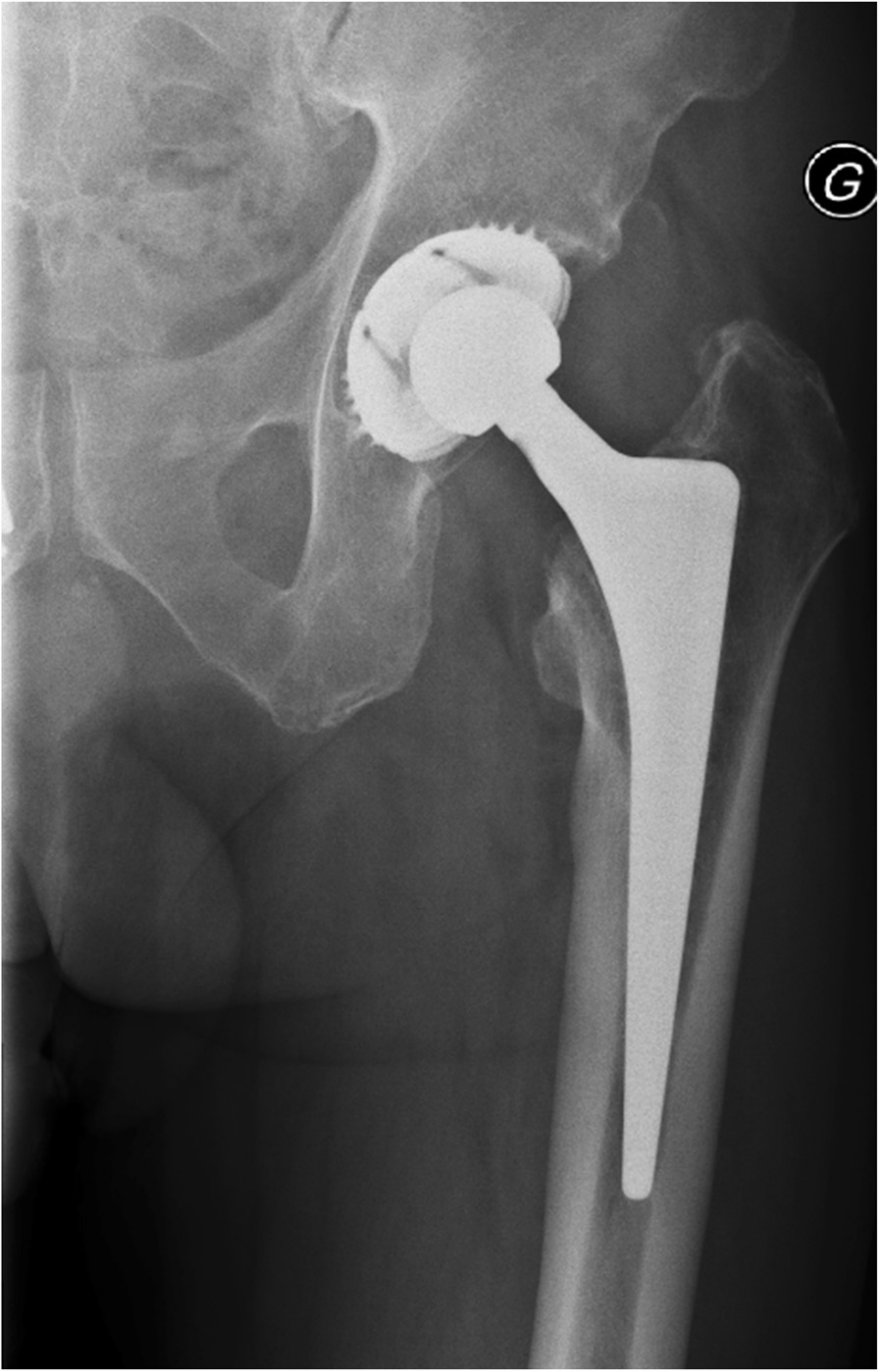
Native x-ray of the hip prosthesis 5 years after surgery. No signs of loosening or migration are visible

At discharge, after completing 4 weeks of intravenous antibiotic treatment, the patient was fully mobilized, reported no pain in the hip and had a normal C-reactive protein value (3 mg/L). Treatment was continued with oral levofloxacin (500 mg every 12 h) plus rifampin (450 mg every 12 h) for additional 2 months to complete the 3 month treatment course. At follow-up visits at 1, 2 and 5 years after revision surgery, the patient presented with a functional, pain-free and radiologically stable hip prosthesis without local signs of infection and with normal serum C-reactive protein. Figure 3 shows the hip x-ray 5 years after surgery.

## Discussion

We present a case of hematogenous infection of hip prosthesis caused by *Granulicatella para-adiacens* occurring 7 years after primary arthroplasty. The infection manifested acutely with fever and discrete local symptoms (joint pain at mobilization and limited range of motion). The most likely origin of the periprosthetic infection was the periapical dental abscess or severe periodontitis. According to modified Duke criteria [28], possible infective endocarditis was diagnosed based on one major criterion (mitral valve vegetation) and two minor criteria: fever and hematogenous seeding of the hip prosthesis. *Granulicatella* is often associated with endocarditis exhibiting affinity to the avascular cordae [29].

Interestingly, *Granulicatella para-adiacens* grew first from the sonication fluid (day 3), followed by the preoperatively aspirated synovial fluid (day 9) and periprosthetic tissue specimens (day 10), indicating the highest density of bacteria on the implant surface [30]. Only one of three periprosthetic tissues grew *Granulicatella para-adiacens* despite presence of visible pus, indicating the importance of sampling several (at least three) periprosthetic tissue specimens during surgery.

To our knowledge, this is the first case of successfully treated implant-associated infection caused by NVS without removal of the fixed components of the hip implant. All previously published cases were treated with a two-stage surgical approach, including an implant-free interval of at least four weeks [12, 17, 19]. In our patient, an acute hematogenous periprosthetic joint infection with an immature biofilm (<3 weeks of age) was diagnosed and only mobile components were exchanged according to current recommendation [31]. After initial intravenous combination of penicillin and gentamicin for 4 weeks (also sufficient for the treatment of possible infective endocarditis), an oral combination therapy with rifampin and levofloxacin was continued in order to eradicate the biofilm on the remaining foreign body.

This case report is illustrative for several reasons. First, it underlines the importance of carrying out proper preoperative and intraoperative procedures (preoperative joint puncture, multiple intraoperative periprosthetic tissue samples, sonication of removed implant parts) to diagnose prosthetic joint infections. Sonication of removed prosthetic parts is particularly useful for rapid and improved microbiological diagnosis, especially when low-burden and patchy distribution of microbial biofilms are expected [30]. Second, joint infections due to NVS occurring after dental work are well described in the literature [17, 32], which underlines the importance of the treatment of periodontitis before implantation of a prosthesis and proper oral hygiene after arthroplasty for primary and secondary prevention of prosthetic joint infection. Third, the NVS may not be needed to be classified as a difficult-to-treat-organism, if exchange of the mobile parts is followed by antimicrobial therapy that also includes biofilm-effective antibiotics such as rifampin. Current guidelines recommend for infective endocarditis due to *Granulicatella* a treatment with antibiotics that are used for treatment of infective endocarditis due to streptococci with intermediate susceptibility to penicillin (combination of penicillins or ceftriaxon or vancomycin 4-6 weeks and gentamicin 2 weeks) [33]. In our case, we performed the combined intravenous treatment for 4 weeks taking in account the difficult-to-treat-character of *Granulicatella* and completed the recommended 12 weeks course of antibiotic therapy for periprosthetic joint infections with streptococci with a combination of levofloxacin and rifampin.

## Conclusion

Although *Granulicatella* is considered a difficult-to-treat bacterial organism, we report successful treatment of a *Granulicatella* hip prosthesis infection and possible infective endocarditis with exchange of mobile parts, debridement and prosthesis retention and a prolonged course of antimicrobial therapy including biofilm-effective antibiotics. The periapical dental abscess is considered the primary focus of hematogenously infected hip prosthesis, underlining the importance treatment of periodontitis prior to arthroplasty and of proper oral hygiene for prevention of hematogenous infection after arthroplasty.

### Ethics and consent to participate

Not applicable.

### Consent to publish

Written informed consent was obtained from the patient for publication of this Case report and any accompanying images.

### Availability of data and materials

All the data supporting our findings is contained within the manuscript.

## Abbreviations

CLSI: clinical and laboratory standards institute
MIC: minimal inhibitory concentration
NVS: nutritionally variant streptococci

## References

[1] Aas JA, Paster BJ, Stokes LN, Olsen I, Dewhirst FE (2005). Defining the normal bacterial flora of the oral cavity. J Clin Microbiol.

[2] Stein DS, Nelson KE (1987). Endocarditis due to nutritionally deficient streptococci: therapeutic dilemma. Rev Infect Dis.

[3] Senn L, Entenza JM, Greub G, Jaton K, Wenger A, Bille J, Calandra T, Prod'hom G. Bloodstream and endovascular infections due to Abiotrophia defectiva and Granulicatella species. BMC Infect Dis. 2006;6:9. 10.1186/1471-2334-6-9 PMC1360077 16426445

[4] Casalta JP, Habib G, La Scola B, Drancourt M, Caus T, Raoult D (2002). Molecular diagnosis of Granulicatella elegans on the cardiac valve of a patient with culture-negative endocarditis. J Clin Microbiol.

[5] Jeng A, Chen J, Katsivas T (2005). Prosthetic valve endocarditis from Granulicatella adiacens (nutritionally variant streptococci). J Infect.

[6] Ohara-Nemoto Y, Kishi K, Satho M, Tajika S, Sasaki M, Namioka A, Kimura S. Infective endocarditis caused by Granulicatella elegans originating in the oral cavity. J Clin Microbiol. 2005;43(3):1405–7. 10.1128/JCM.43.3.1405-1407.2005 PMC1081294 15750118

[7] Brouqui P, Raoult D (2001). Endocarditis due to rare and fastidious bacteria. Clin Microbiol Rev.

[8] Namdari H, Kintner K, Jackson BA, Namdari S, Hughes JL, Peairs RR, Savage DJ. Abiotrophia species as a cause of endophthalmitis following cataract extraction. J Clin Microbiol. 1999;37(5):1564–6. 10.1128/jcm.37.5.1564-1566.1999 PMC84829 10203522

[9] Keay L, Harmis N, Corrigan K, Sweeney D, Willcox M (2000). Infiltrative keratitis associated with extended wear of hydrogel lenses and Abiotrophia defectiva. Cornea.

[10] Cerceo E, Christie JD, Nachamkin I, Lautenbach E (2004). Central nervous system infections due to Abiotrophia and Granulicatella species: an emerging challenge?. Diagn Microbiol Infect Dis.

[11] Finegold SM, Flynn MJ, Rose FV, Jousimies-Somer H, Jakielaszek C, McTeague M, Wexler HM, Berkowitz E, Wynne B. Bacteriologic findings associated with chronic bacterial maxillary sinusitis in adults. Clin Infect Dis. 2002;35(4):428–33. 10.1086/341899 12145727

[12] Riede U, Graber P, Ochsner PE (2004). Granulicatella (Abiotrophia) adiacens infection associated with a total knee arthroplasty. Scand J Infect Dis.

[13] Adam EL, Siciliano RF, Gualandro DM, Calderaro D, Issa VS, Rossi F, Caramelli B, Mansur AJ, Strabelli TM. Case series of infective endocarditis caused by Granulicatella species. Int J Infect Dis. 2015;31:56–8. 10.1016/j.ijid.2014.10.023 25461651

[14] Ruoff KL (1991). Nutritionally variant streptococci. Clin Microbiol Rev.

[15] Habib G, Hoen B, Tornos P, Thuny F, Prendergast B, Vilacosta I, Moreillon P, de Jesus Antunes M, Thilen U, Lekakis J et al. Guidelines on the prevention, diagnosis, and treatment of infective endocarditis (new version 2009): the Task Force on the Prevention, Diagnosis, and Treatment of Infective Endocarditis of the European Society of Cardiology (ESC). Endorsed by the European Society of Clinical Microbiology and Infectious Diseases (ESCMID) and the International Society of Chemotherapy (ISC) for Infection and Cancer. Eur Heart J. 2009;30(19):2369–413. 10.1093/eurheartj/ehp285 19713420

[16] Ince A, Tiemer B, Gille J, Boos C, Russlies M (2002). Total knee arthroplasty infection due to Abiotrophia defectiva. J Med Microbiol.

[17] Mougari F, Jacquier H, Bercot B, Hannouche D, Nizard R, Cambau E, Zadegan F. Prosthetic knee arthritis due to Granulicatella adiacens after dental treatment. J Med Microbiol. 2013;62(Pt 10):1624–7. 10.1099/jmm.0.058263-0 23764743

[18] Cassir N, Grillo JC, Argenson JN, Drancourt M, Levy PY (2011). Abiotrophia defectiva knee prosthesis infection: a case report. J Med Case Rep.

[19] Rozemeijer W, Jiya TU, Rijnsburger M, Heddema E, Savelkoul P, Ang W (2011). Abiotrophia defectiva infection of a total hip arthroplasty diagnosed by 16S rRNA gene sequencing. Diagn Microbiol Infect Dis.

[20] del Pozo JL, Garcia-Quetglas E, Hernaez S, Serrera A, Alonso M, Pina L, Leiva J, Azanza JR. Granulicatella adiacens breast implant-associated infection. Diagn Microbiol Infect Dis. 2008;61(1):58–60. 10.1016/j.diagmicrobio.2007.12.009 18206331

[21] Frenkel A, Hirsch W (1961). Spontaneous development of L forms of streptococci requiring secretions of other bacteria or sulphydryl compounds for normal growth. Nature.

[22] Kanamoto T, Sato S, Inoue M (2000). Genetic heterogeneities and phenotypic characteristics of strains of the genus Abiotrophia and proposal of Abiotrophia para-adiacens sp. nov. J Clin Microbiol.

[23] Collins MD, Lawson PA (2000). The genus Abiotrophia (Kawamura et al.) is not monophyletic: proposal of Granulicatella gen. nov., Granulicatella adiacens comb. nov., Granulicatella elegans comb. nov. and Granulicatella balaenopterae comb. nov. Int J Syst Evol Microbiol.

[24] Cargill JS, Scott KS, Gascoyne-Binzi D, Sandoe JA (2012). Granulicatella infection: diagnosis and management. J Med Microbiol.

[25] Jorgensen N, James H, Turnidge JD (2015). Susceptibility test methods: dilution and disk diffusion methods.

[26] Rosenthal O, Woywodt A, Kirschner P, Haller H (2002). Vertebral osteomyelitis and endocarditis of a pacemaker lead due to Granulicatella (Abiotrophia) adiacens. Infection.

[27] Bemer P, Plouzeau C, Tande D, Leger J, Giraudeau B, Valentin AS, Jolivet-Gougeon A, Vincent P, Corvec S, Gibaud S et al. Evaluation of 16S rRNA gene PCR sensitivity and specificity for diagnosis of prosthetic joint infection: a prospective multicenter cross-sectional study. J Clin Microbiol. 2014;52(10):3583–9. 10.1128/JCM.01459-14 PMC4187742 25056331

[28] Li JS, Sexton DJ, Mick N, Nettles R, Fowler Jr VG, Ryan T, Bashore T, Corey GR. Proposed modifications to the Duke criteria for the diagnosis of infective endocarditis. Clin Infect Dis. 2000;30(4):633–8. 10.1086/313753 10770721

[29] Senn L, Entenza JM, Prod'hom G (2006). Adherence of Abiotrophia defectiva and Granulicatella species to fibronectin: is there a link with endovascular infections?. FEMS Immunol Med Microbiol.

[30] Trampuz A, Piper KE, Jacobson MJ, Hanssen AD, Unni KK, Osmon DR, Mandrekar JN, Cockerill FR, Steckelberg JM, Greenleaf JF et al. Sonication of removed hip and knee prostheses for diagnosis of infection. N Engl J Med. 2007;357(7):654–63. 10.1056/NEJMoa061588 17699815

[31] Zimmerli W, Trampuz A, Ochsner PE (2004). Prosthetic-joint infections. N Engl J Med.

[32] Taylor CE, Fang MA (2006). Septic arthritis caused by Abiotrophia defectiva. Arthritis Rheum.

[33] Habib G, Lancellotti P, Antunes MJ, Bongiorni MG, Casalta JP, Del Zotti F, Dulgheru R, El Khoury G, Erba PA, Iung B et al. 2015 ESC Guidelines for the management of infective endocarditis: The Task Force for the Management of Infective Endocarditis of the European Society of Cardiology (ESC). Endorsed by: European Association for Cardio-Thoracic Surgery (EACTS), the European Association of Nuclear Medicine (EANM). Eur Heart J. 2015;36(44):3075–128. 10.1093/eurheartj/ehv319 26320109

